# Applying ethical dimensions in clinical dilemmas

**DOI:** 10.1192/bjo.2021.442

**Published:** 2021-06-18

**Authors:** Rachel Swain, Kazeem Owudunni, Graham Behr, Jo Emmanuel, Matt Malherbe

**Affiliations:** 1 West London NHS Trust; 2 Central and North West London NHS Foundation Trust

## Abstract

**Aims:**

Central and North West London's Clinical Ethics Committee (CEC) offers a non-judgmental space to discuss ethical concerns and challenges and provide ethical guidance. This project aims to publicise these ethical dilemmas and guidance to inform decision making trust-wide.

**Background:**

A Clinical Ethics Committee (CEC) encompasses a diverse range of figures, from psychiatrists and general practitioners to members of the clergy and experts by experience. The CEC in Central and North West London have been meeting regularly since 2003 to provide ethical assistance to a wide range of medical, surgical and psychiatric teams. Complex ethical cases are presented by the treating team, allowing a subsequent discussion of the ethical theories and frameworks within the case with the committee members. This synthesis of information can then assist the treating team in the shaping of ethical based solutions to their dilemmas.

The committee wished to encourage ethical based clinical thinking within the trust and enable others to learn from the valuable insights already provided by the CEC over the years.

**Method:**

Case notes, recorded from the last 17 years of meetings of the Clinical Ethics Committee were reviewed. 98 cases were identified between 2003-2019. The contemporaneous case reports were then anonymised and indexed into one easy to use file. This file was published on the local intranet and publicised to staff.

**Result:**

The cases were compiled into a PDF document which is available for all staff members within the trust on the intranet. This resource is open to all clinical staff, and serves the dual purpose of encouraging ethical-based thinking and also promoting the ethics committee to those who might be in need of assistance.

**Conclusion:**

Clinical decisions can be complex and nuanced, often complicated by multiple viewpoints and ways of thinking. The database demonstrates the use of ethical dimensions by the ethics committee to inform decision making in a series of varied clinical and management dilemmas. The project required careful consideration around preservation of confidentiality as well as overcoming the logistical barriers of trust-wide dissemination. The result is a document that will allow ethical based decision-making to be embedded into everyday practice.

